# Application of CT pulmonary angiography and echocardiography in acute pulmonary embolism: A cross‐sectional study

**DOI:** 10.1002/hsr2.1546

**Published:** 2023-09-04

**Authors:** Mozhgan Sametzadeh, Sahar Dadgostar, Mohammad Ghasem Hanafi, Mohammad Mohammadi

**Affiliations:** ^1^ Department of Radiology, School of Medicine, Golestan Hospital Ahvaz Jundishapur University of Medical Sciences Ahvaz Iran; ^2^ Department of Radiology, School of Medicine Ahvaz Jundishapur University of Medical Sciences Ahvaz Iran; ^3^ Department of Radiology, School of Medicine, Imam Khomeini Hospital Ahvaz Jundishapur University of Medical Sciences Ahvaz Iran; ^4^ Department of Cardiology, School of Medicine, Golestan Hospital Ahvaz Jundishapur University of Medical Sciences Ahvaz Iran

**Keywords:** angiography, computed tomography, diagnosis, echocardiography, pulmonary embolism

## Abstract

**Background:**

Pulmonary Embolism (PE) is an acute and potentially fatal condition defined as the blockage of pulmonary arteries by an embolism that can be from various origins.

**Objective:**

The present study aimed to investigate the findings of computed tomography pulmonary angiography (CTPA) and echocardiography in patients with acute PE.

**Methods:**

The present cross‐sectional study included some patients with clinical manifestations of PE who underwent CTPA and echocardiography. The radiologic findings, PE severity, and outcome of the patients were recorded. Moreover, echocardiography was performed by an expert cardiologist using a high‐resolution device, while CTPA was performed by an expert radiologist using a 16‐slice device and a two‐step selective test bolus method.

**Results:**

According to our findings, a total number of 147 patients were diagnosed with PE, including 44 (29.93%), 44 (29.93%), and 59 (40.14%) cases of mild, moderate, and severe PE, respectively. Moreover, 25 patients (17%) finally expired due to PE. Regarding the CTPA findings, 31 patients (21.1%) had septum flattening, while 35 (23.8%) had a septum deviation toward the left ventricle. Also, there were significant correlations between mortality and some CTPA findings, including severe PE (*p* < 0.001), the presence of septal deviation (*p* = 0.007), and higher diameters of the main pulmonary artery (*p* < 0.001) and right ventricle (*p* = 0.008).

**Conclusion:**

CTPA is a valid and accessible modality for diagnosing and evaluating PE in suspected patients. Moreover, several findings in CTPA could predict adverse outcomes, such as death, in patients with PE.

## Key points

- CTPA can help diagnose and treat pulmonary embolism early.
- This modality may have life‐threatening consequences.

## 1 INTRODUCTION

Pulmonary embolism (PE) is an acute and potentially fatal condition defined as the blockage of pulmonary arteries by an embolism that can be from various origins, mainly a deep vein thrombosis (DVT) in the lower extremities.^1^, ^2^ According to recent studies, symptomatic PE has an annual incidence of 0.5–1 per 1000 individuals,^3^ with a sharp rise in the incidence in both men and women after the age of 60.^4^ The development of PE is associated with several risk factors, including inherited (such as the dysfunction of the blood coagulation factors), acquired (such as trauma, major surgery, malignancy, obesity, and pregnancy), and mixed factors.^1^ Moreover, several pharmacological and nonpharmacological interventions are used for PE prophylaxis and treatment.^5^, ^6^

The diagnosis of PE is made based on clinical findings (Wells' Criteria), laboratory investigations, and imaging findings.^3^ About two decades ago, PE was noninvasively diagnosed using echocardiography.^7^, ^8^ However, more advanced modalities, including computed tomography pulmonary angiography (CTPA) and ventilation‐perfusion (VQ) scan, are currently the most commonly used imaging modalities for PE in several healthcare facilities.^3^ Among these two, CTPA is the gold standard for diagnosing PE, considering its high accuracy in differentiating PE and other pathologies.^3^, ^9^ However, some disadvantages have been reported for this modality that limit its routine use, including considerable radiation exposure, a potential allergic reaction to iodine contrasts, contrast‐induced acute kidney injury, and device‐related limitations in obese patients.^3^, ^9^

According to evidence, the findings of echocardiography and CTPA highly correlated in diagnosing and evaluating acute PE.^10^, ^11^ Regarding the lower availability and the specific disadvantages of CTPA, it would be beneficial if other imaging modalities with higher availability, such as echocardiography, could be used as the routine imaging modality for PE diagnosis. However, such a measure necessitates further studies and a thorough comparison between echocardiography and CTPA to resolve the current controversies and prove the efficacy of CTPA or echocardiography in PE diagnosis. Therefore, the present study aimed to investigate the findings of CTPA and echocardiography in some patients with PE.

## 2 MATERIALS AND METHODS

### 2.1 Study design and population

This was a cross‐sectional study to explore the aim of this investigation. Patients with clinical symptoms suspicious of acute PE were recruited in this study, during two years of 2019 and 2020, in the Golestan hospitals of Ahvaz City, Iran. Criteria for inclusion in this study were (#1) clinical suspicion of acute PE based on the American College of Radiology (ACR),^12^ (#2) age >18‐year‐old, (#3) acceptable quality of CTPA, and (#4) consenting to the study. Exclusion criteria from this study were (#1) age <18‐year‐old, (#2) history of chronic PE, (#3) history of known cardiovascular disease, (#3) PE was not diagnosed by CTPA, and (#5) patient's preference to not participating in a study for any reason and consent withdrawal.

### 2.2 Imaging protocol

Echocardiography evaluation was done by an expert cardiologist with a high‐resolution device (Philips, EPIQ 7 Ultrasound Machine). CTPA was done under the supervision of an expert radiologist with a 16‐slices device (Siemens, Somatom emotion) (voltage 100–120 kVP, pitch 1.5, rotation time 0.6 s, detector width 16 × 0.6 mm) through a two‐step selective test bolus method. In the first step as the test step, 15–30 cc of the Visipaque (iodixanol injectable contrast medium for intravascular use) contrast (350 mg/cc) was injected with a flow rate of 5–6 cc/s and multiple scans were done from the pulmonary artery bifurcation. By the test injection, the delay time was calculated by the dynaeva option of the device, and the delay time was used for the imaging protocol of the main step with an injection of 70–90 cc of the contrast with the same flow rate and the scans were obtained from the pulmonary artery. The results of CTPA were reported by the radiologist regardless of the echocardiography and the clinical status of the patients.

### 2.3 Study variables

The main study variables were radiologic findings in the CTPA evaluation including the severity of PE (mild/moderate/severe), main pulmonary artery diameter (mm), aorta diameter (mm), right ventricle diameter (mm), left ventricle diameter (mm), septal deviation (negative/septum flattening/septum deviate toward left ventricle), wedge consolidation (absent/present), inferior vena cava (IVC) reflux (absent/present), and pulmonary trunk volume (mm^3^). The variables investigated by echocardiography were pulmonary artery pressure (mmHg), septal deviation (absent/present), right ventricle enlargement (absent/present), and left ventricle enlargement (absent/present). The main clinical variable was the patients' outcome (expired/alive) during the course of the study.

### 2.4 Sample size

Using Cochran's formula and based on a literature review the sample size was estimated at 159 participants.^13^

$$n_{0}=Z_{1-\alpha /2}^{2}\times p\times \left(1-p\right)/d^{2}.$$

### 2.5 Statistical analysis

The descriptive presentation of the results included the mean and standard deviation (SD) of the quantitative variables and the frequency and percentage of the qualitative variables. The *χ* ^2^ test and independent student *t* test were used to statistically explore the findings between variables. Fisher's exact test was used as the statistical significance test in analyzing contingency tables with small sample sizes. *p* < 0.05 were assumed as the significance level. All statistical analyses were conducted by the IBM SPSS Statistics package (SPSS for Windows, Version 22.0; SPSS Inc.).

## 3 RESULTS

A total number of 147 patients were included in this study. The severity of diagnosed PE was mild in 44 (29.93%) patients, moderate in 44 (29.93%) cases, and severe in 59 (40.14%) cases. Finally, 25 (17.0%) patients expired due to PE in this study and 122 (83.0%) cases survived the condition.

**Figure 1:**
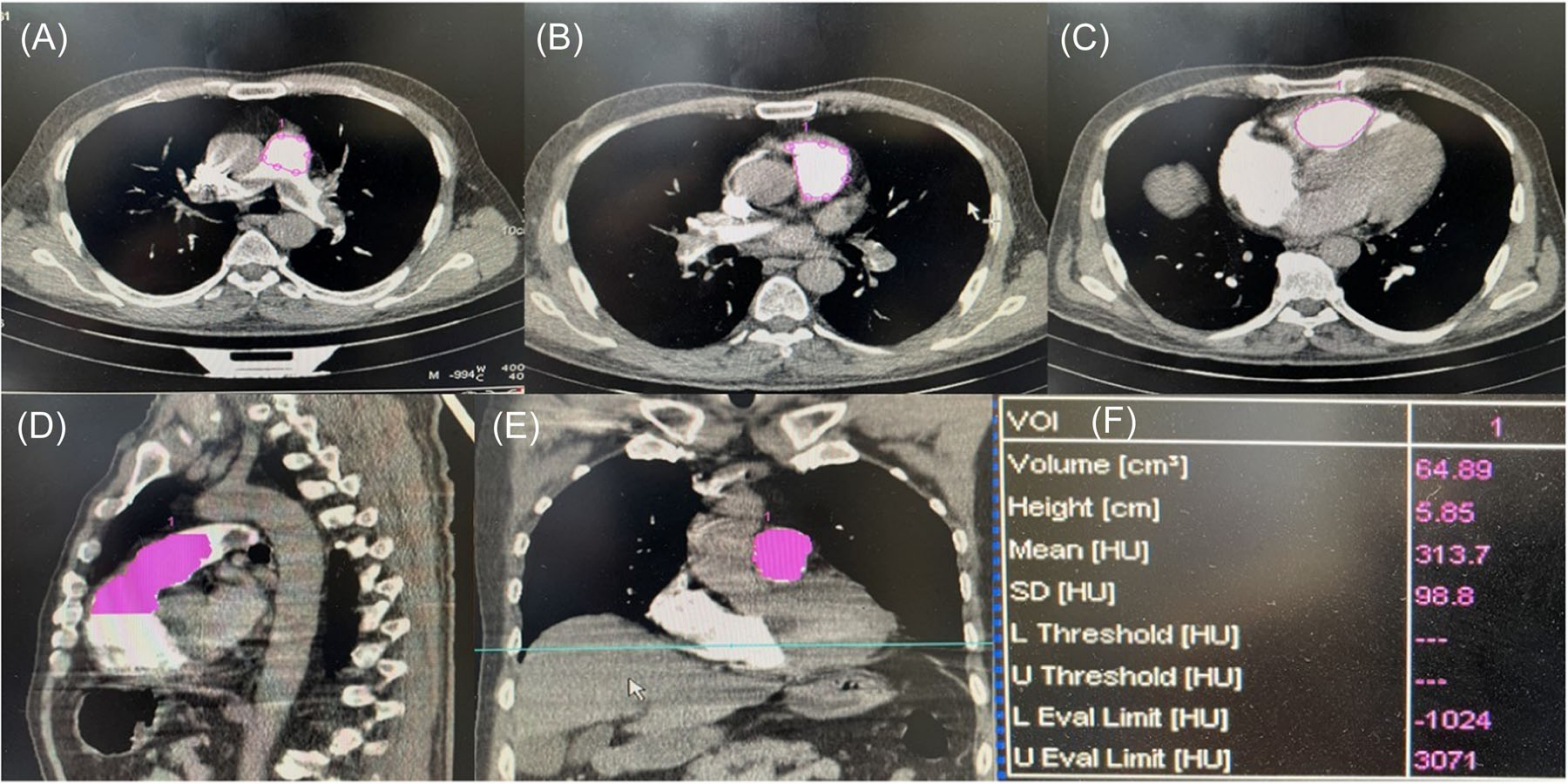
The process of evaluation of pulmonary artery trunk volume in CTPA by SOMATOM Emotion software of the CT device. In these images, axial cut of pulmonary artery trunk is shown in in three planes of pulmonary bifurcation (A), median part of pulmonary trunk (B), and the initial part of pulmonary trunk just after the tricuspid valve (C) which are delignated by pink lines. The software calculates the pulmonary trunk volume the marked lines (D and E) and generates the values in a table (F).

CTPA findings showed negative septal deviation in 81 (55.1%) cases, septum flattening in 31 (21.1%) cases, and septum deviation toward the left ventricle in 35 (23.8%) patients. Wedge consolidation was present in 49 (33.3%) cases and the IVC reflux was seen in 46 (31.3%) patients. The mean diameter of the main pulmonary artery was 28.8 mm (SD: 4.5, range: 20–47.5). The mean diameter of the aorta was 32.7 mm (SD: 5.2, range: 20–56). The mean diameter of the right and left ventricles in this evaluation were 40.3 mm (SD: 8.1, range: 20–62) and 39.3 mm (SD: 7.7, range: 13–72), respectively. Also, the mean pulmonary trunk volume was 52.4 mm^3^ (SD: 16.3, range: 23–101). The steps of evaluation of pulmonary artery trunk volume are depicted in Figure 1.

This result shows a 6.94% missing values for echocardiography data (Table 1). The echocardiography evaluation showed septal deviation in one (0.68%) patient, right ventricle enlargement in 31 (21.08%) patients, and left ventricle enlargement in seven (4.76%) patients. The mean pulmonary artery pressure in echocardiography was 35.7 mmHg (SD: 14.5, range: 18–78).

**Table 1:** The results depicting comparisons of patients who died to those alive.

Variables		Mortality		% of missing values	*p* Value
		Yes (*N* = 25)	No (*N* = 122)		
The severity of PE, *N* (%)	Mild	3 (12)	41 (33.6)	0.00	<0.001*
	Moderate	2 (8)	42 (34.4)		
	Severe	20 (80)	39 (32)		
CTPA findings, *N* (%)	Negative septal deviation	10 (40)	71 (58.2)	0.00	0.007*
	Septum flattening	3 (12)	28 (23)		
	Septum deviation toward left ventricle	12 (48)	23 (18.9)		
Wedge consolidation, *N* (%)		5 (20)	44 (36.1)	0.00	0.121
IVC reflux, *N* (%)		10 (40)	36 (29.5)	0.00	0.303
The mean diameter of the main pulmonary artery (mm), mean ± SD (range)		32.2 ± 5.1 (24–47.5)	28.12 ± 4.01 (20–38)	0.00	<0.001**
The mean diameter of right/left ventricles(mm), mean ± SD (range)		44.21 ± 8.15 (31–60)/39.84 ± 10.55 (13–60)	39.52 ± 7.94 (20–62)/39.24 ± 7.09 (24‐72)	0.00	0.008**/0.791
The mean pulmonary trunk volume (mm^3^), mean ± SD (range)		66 ± 16.58 (33–101)	49.63 ± 14.77 (23–94)	0.00	<0.001**
The echocardiography evaluation	Septal deviation	0	1 (0.8)	6.94	0.99
	Right ventricle enlargement	5 (20)	26 (21.3)		
	Left ventricle enlargement	0	7 (5.7)		
The mean pulmonary artery pressure in echocardiography (mmHg), mean ± SD (range)		46.7 ± 14.96 (27–78)	34.27 ± 13.9 (18–75)	0.00	<0.001**

Abbreviation: PE, pulmonary embolism.

* Kruskal‐Wallis.

** Mann‐Whitney.

The severity of PE in CTPA was significantly correlated with the mortality in patients in this study which happened in 12% of mild cases, 8% of moderate cases, and 80% of severe cases (*p* < 0.001). Also, the septal deviation was significantly correlated with mortality as the outcome happened in 40% of patients without deviation, 12% of patients with septal flattening, and 48% of patients with deviated septum toward the left ventricle (*p* = 0.007). In contrast, patients with wedge consolidation had lower mortality (20%), but the association was not significant (*p* = 0.121). Also, mortality happened more in patients with IVC reflux (40%), but this was not also statistically significant (*p* = 0.303). The mean diameter of the main pulmonary artery was larger in CTPA in patients who expired due to PE (*p* < 0.001). The mean diameter of the aorta was not significantly different in this evaluation between expired and alive patients (*p* = 0.485). The mean diameter of the right ventricle was considerably larger in expired patients (*p* = 0.008). However, the mean diameter of the left ventricle was not associated with expiration due to PE (*p* = 0.791). The mean pulmonary trunk volume was significantly larger in expired patients (*p* < 0.001) (Table 1).

**Figure 2:**
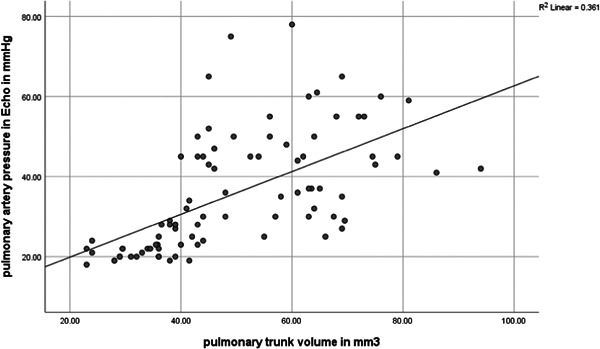
Scatter plot of pulmonary trunk volume in CTPA and pulmonary artery pressure in echocardiography. CTPA, computed tomography pulmonary angiography.

Regarding the findings of echocardiography, the findings on septal deviation, right ventricle enlargement, and left ventricle enlargement were not significantly associated with the mortality outcome (*p* = 0.99). Although, the mean pulmonary artery pressure in echocardiography was higher in expired patients due to PE. Regarding the evaluated pulmonary artery trunk volume in CTPA and the estimated pulmonary artery pressure in echocardiography, the values of these two variables were significantly correlated; however, the correlation was medium in strength (Pearson's correlation coefficient: 0.601, *p* < 0.001) (Figure 2, Table 1).

## 4 DISCUSSION

According to our findings, most patients had moderate or severe PE. Moreover, the mortality rate of PE was about 17% in our patients. Also, various cardiac and pulmonary features of PE were observed using CTPA and echocardiography, and there were significant correlations between mortality and some CTPA‐related markers, including the severity of PE, presence of septal deviation, the size of the pulmonary trunk, and the higher diameters of the main pulmonary arteries and right ventricle. Finally, our findings showed the effectiveness of CTPA in predicting adverse outcomes, such as mortality, in patients with PE. Therefore, the findings of the present study highlighted the role of CTPA as the first‐line imaging modality for the diagnosis and management of patients with PE.

A similar study in Iran investigated the patients with suspected PE using clinical methods, echocardiography, and CTPA, showing that of all patients with suspected PE who underwent CTPA, only a few received a confirmed PE diagnosis. The authors recommended using thorough criteria for choosing the candidates for CTPA since this modality is more effective in highly suspicious patients, and its application to all patients may be unnecessary and lead to excessive radiation exposure and other complications of CTPA.^14^

Moreover, there have been other controversial reports on the overuse of CTPA for PE diagnosis. Another study on this topic reported that of all patients undergoing CTPA for suspected PE, the condition was only confirmed in a few patients. Therefore, the authors stated the overuse of CTPA for PE diagnosis and suggested using an improved algorithm for choosing CTPA candidates to cut its unnecessary use.^15^ On the other hand, a study showed that the presence of right ventricular strain in CTPA was an accurate predictor of PE‐related mortality during hospitalization.^16^ However, another study on this topic showed that in most cases, signs of right ventricle strain in CTPA do not reliably predict the severity of PE and subsequent adverse clinical outcomes. Therefore, CTPA should be used with caution in patients with suspected acute PE so the chance of thrombolytic use is not missed due to delays for additional imaging.^17^ Moreover, some studies have shown that even in the cases in which PE is excluded, the cardiovascular data from CTPA can be used for diagnosing other pathologies, such as pulmonary hypertension.^18^ Nevertheless, there is a need for additional studies for clarifying these ambiguities and providing strong guidelines for the use of CTPA in PE diagnosis.

According to the present study's findings, CTPA is an accurate and valid imaging modality that can be used as the first‐line choice for evaluating patients with suspected PE. However, it should not be used in all cases with suspected PE, and the choice of patients for CTPA should be individualized to minimize the chance of complications and unnecessary radiation exposure. Moreover, it is highly recommended to optimize the CTPA quality, time of use, contrast concentration and injection flow rate, and dose of radiation to maximize the benefits of this imaging modality for the patients.^19^

The present study evaluated the findings of echocardiography and CTPA in patients with PE. Although we made a more comprehensive evaluation of the CTPA features, the findings by echocardiography were almost consistent with those of CTPA. Moreover, a recent large‐scale study comparing the findings of echocardiography and CTPA in patients with PE raised two points.^10^, ^11^ First, the presence of right ventricular strain in echocardiography and an increased ratio of right ventricle diameter to left ventricle diameter was a predictor of PE‐related 30‐day mortality with almost similar prognostic significance,^10^ and second, the findings of CTPA and echocardiography moderately correlated on the right ventricular size in acute PE.^11^

In recent years, novel integrated measures consisting of imaging and laboratory investigations have been introduced for risk stratification and patient management in acute PE. For instance, a combination of automated 3D volumetry of central pulmonary arteries using CTPA findings and pressure estimations using echocardiography is used for the noninvasive diagnosis of pulmonary hypertension.^20^ Surprisingly, a study proposed that serum NT‐proBNP and troponin T levels can be superior to the combination of biomarkers and right ventricular dysfunction findings in CTPA/echocardiography in risk stratification of patients with normotensive PE.^21^ Moreover, one of the well‐known methods is the CT index, which quantifies the arterial obstruction in PE and highly correlates with CTPA findings.^22^, ^23^, ^24^ Further expansion of these noninvasive and operator‐independent methods could help in the early diagnosis of PE and the successful prediction of its adverse outcomes.

The present study had some limitations as well, including the small sample size. It is recommended to perform further studies on this topic using larger sample sizes to include more diverse findings in the imaging. Moreover, we did not provide any data on other routine imaging evaluations used for PE diagnosis, such as VQ scan, as well as laboratory findings. Furthermore, this was a retrospective study and 6.94% of patients expired before doing echocardiography; therefore, this data was missed. Also, the present study did not investigate the interval between the diagnosis and mortality in the deceased patients. However, despite all limitations, the present study could report the CTPA and echocardiography findings in a sample of Iranian patients with PE. The dual comparison of these findings was the major strength of the present study.

## 5 CONCLUSION

According to the present study's findings, CTPA is a valid and accessible modality for diagnosing and evaluating PE in suspected patients. Moreover, several findings in CTPA can predict adverse outcomes, such as death, in patients with PE. The findings of the present study can help in early diagnosis and treatment of PE, thereby preventing life‐threatening consequences.

## AUTHOR CONTRIBUTIONS

**Mozhgan Sametzadeh**: Project administration; supervision; writing—original draft. **Sahar Dadgostar**: Methodology; resources; writing—original draft. **Mohammad Ghasem Hanafi**: Data curation; writing—review & editing. **Mohammad Mohammadi**: Methodology; resources.

## CONFLICT OF INTEREST STATEMENT

The authors declare no conflict of interest.

## ETHICAL STATEMENT

The study obtained the approval of the institutional review board at Golestan Medical and Research Center (code: IR.AJUMS.HGOLESTAN.REC.1399.104). This study used recorded data of patients and no intervention was done on included population. All patients provided informed consent before participation in this study.

## TRANSPARENCY STATEMENT

The lead author Sahar Dadgostar affirms that this manuscript is an honest, accurate, and transparent account of the study being reported; that no important aspects of the study have been omitted; and that any discrepancies from the study as planned (and, if relevant, registered) have been explained.

## Data Availability

Data available on request from the authors.
